# Predicting the Development of Renal Replacement Therapy Indications by Combining the Furosemide Stress Test and Chemokine (C-C Motif) Ligand 14 in a Cohort of Postsurgical Patients

**DOI:** 10.1097/CCM.0000000000005849

**Published:** 2023-03-29

**Authors:** Melanie Meersch, Raphael Weiss, Joachim Gerss, Felix Albert, Janik Gruber, John A. Kellum, Lakhmir Chawla, Lui G. Forni, Jay L. Koyner, Thilo von Groote, Alexander Zarbock

**Affiliations:** 1 Department of Anesthesiology, Intensive Care Medicine and Pain Medicine, University Hospital Münster, Münster, Germany.; 2 Institute of Biostatistics and Clinical Research Medical Faculty, University of Münster, Münster, Germany.; 3 The Center for Critical Care Nephrology, CRISMA, Department of Critical Care Medicine, University of Pittsburgh, Pittsburgh, PA.; 4 Veterans Affairs Medical Center, San Diego, CA.; 5 Department of Intensive Care Medicine, Royal Surrey Hospital and Faculty of Health Sciences, University of Surrey, Surrey, United Kingdom.; 6 Section of Nephrology, Department of Medicine, University of Chicago, Chicago, IL.

**Keywords:** acute kidney injury, biomarkers, furosemide stress test, renal replacement therapy

## Abstract

Optimal timing of renal replacement therapy (RRT) initiation in severe acute kidney injury (AKI) remains controversial. Initiation of treatment early in the course of AKI may lead to some patients undergoing unnecessary RRT, whereas delayed treatment is associated with increased mortality. This study aims to investigate whether the combination of the furosemide stress test (FST) and AKI-associated biomarkers can predict the development of indications for RRT.

**DESIGN:**

Single-center, prospective, observational study.

**SETTING:**

University Hospital of Muenster, Germany.

**PATIENTS:**

Critically ill, postoperative patients with moderate AKI (Kidney Disease: Improving Global Outcomes stage 2) and risk factors for further progression (vasopressors and/or mechanical ventilation) receiving an FST.

**INTERVENTIONS:**

Sample collection and measurement of different biomarkers (chemokine [C-C motif] ligand 14 [CCL14], neutrophil gelatinase-associated lipocalin, dipeptidyl peptidase 3).

**MEASUREMENT AND MAIN RESULTS:**

The primary endpoint was the development of greater than or equal to one predefined RRT indications (hyperkalemia [≥ 6 mmol/L], diuretic-resistant hypervolemia, high urea serum levels [≥ 150 mg/dL], severe metabolic acidosis [pH ≤ 7.15], oliguria [urinary output < 200 mL/12 hr], or anuria). Two hundred eight patients were available for the primary analysis with 108 having a negative FST (urine output < 200 mL in 2 hr following FST). Ninety-eight patients (47%) met the primary endpoint, 82% in the FST negative cohort. At the time of inclusion, the combination of a negative FST test and high urinary CCL14 levels had a significantly higher predictive value for the primary endpoint with an area under the receiver operating characteristic curve (AUC) of 0.87 (95% CI, 0.82–0.92) compared with FST or CCL14 alone (AUC, 0.79; 95% CI, 0.74–0.85 and AUC, 0.83; 95% CI, 0.77–0.89; *p* < 0.001, respectively). Other biomarkers showed lower AUCs.

**CONCLUSIONS:**

The combination of the FST with the renal biomarker CCL14 predicts the development of indications for RRT.

**KEY POINTS**

**Question:** Single-center, prospective study in Germany, including 208 critically ill patients with acute kidney injury (AKI) receiving a furosemide stress test (FST) to predict an absolute indication for renal replacement therapy (RRT).

**Findings:** The combination of a negative FST test and high urinary biomarker chemokine (C-C motif) ligand 14 (CCL14) had a significantly higher predictive value for the primary endpoint with an area under the receiver operating characteristic curve of 0.87 (95% CI, 0.82–0.92) compared with FST or CCL14 alone.

**Meanings:** The combination of FST and CCL14 may be a promising tool to individualize prognostication and therapy in critically ill patients with AKI in regards to RRT.

Acute kidney injury (AKI) is a common and serious complication in critically ill patients affecting nearly half of patients in the ICU with 10% requiring renal replacement therapy (RRT) (1). AKI significantly affects morbidity and mortality with patients developing severe AKI with life-threatening complications having especially poor outcomes. RRT remains the mainstay of management of severe AKI but the optimal timing remains controversial. Several randomized controlled trials have shown that the use of RRT in critically ill patients with AKI is highly variable and that starting RRT based on AKI severity results in over 40% of patients who spontaneously recover from AKI not requiring RRT while those receiving it late have higher mortality (2–4). However, there is no established definition of “early” and “late,” highlighting the need for better tools to define the optimal timing of initiation.

The furosemide stress test (FST) has been evaluated for predicting AKI progression (5), showing that low urine output following FST had a high predictive value for AKI progression to more severe stages of AKI (5). One trial used the FST test to predict the need for RRT, but this pilot feasibility trial was negative (6). A further option for detecting AKI progression may be the use of novel biomarkers. In the past, neutrophil gelatinase-associated lipocalin (NGAL), kidney injury molecule 1 (KIM-1), and interleukin-18 (IL-18) have been evaluated for predicting RRT. In a meta-analysis, the area under the receiver operating characteristic curves (AUCs) for predicting RRT were 0.720 (95% CI, 0.630–0.803), 0.722 (0.575–0.868), and 0.668 (0.606–0.729) for NGAL, KIM-1, and IL-18, respectively (7). However, heterogeneity was very high as the indications for initiation were variable. Recently, a new biomarker urinary chemokine (C-C motif) ligand 14 (CCL14) has been shown to predict persistence of AKI in patients with moderate AKI (8). The AUC for predicting persistent AKI was 0.83 (0.78–0.87) in the critical care setting and 0.915 (95% CI, 0.858–0.972) in the cardiac surgery setting (8, 9). Importantly, not every patient developing a persistent or severe AKI (Kidney Disease: Improving Global Outcomes [KDIGO] stage 3) develops widely accepted indications for RRT (10).

This prospective, observational, single-center trial was designed to evaluate whether the combination of two strategies (FST test and novel biomarkers) may identify those patients who ultimately develop an indication for RRT.

## METHODS

### Study Design and Ethics

This was a single-center observational trial conducted between August 2018 and May 2022 at the University Hospital Münster. Institutional review board approval was obtained by the Research Ethics Committee of the Chamber of Physicians Westfalen-Lippe and the Westfalian Wilhelms University Muenster (2019-261-f-S) on May 7, 2019. The study was conducted in accordance with the Declaration of Helsinki (Version Fortaleza, 2013). Written informed consent was obtained from all participating patients according to local requirements and legislation.

### Participants

Critically ill adult patients with an oliguric stage 2 AKI were included in the study (10). At study inclusion, patients had to be either mechanically ventilated and/or receiving vasopressors (norepinephrine/epinephrine/norepinephrine + epinephrine ≥ 0.1 µg/kg/min) to be eligible. Patients were excluded if any or several of the following conditions was met: advanced chronic kidney disease (CKD) with estimated glomerular filtration rate less than 20 mL/min/1.73 m^2^, chronic dialysis dependency, need for RRT due to drug intoxication, pregnancy or breastfeeding, or participation in another interventional trial within the last 30 days.

### Study Procedures

Prior to performing the FST, hypovolemia was excluded (by passive leg raising test or echocardiography) or treated and blood and urine samples were collected. FST was performed IV using 1.0 mg/kg in diuretic-naive and 1.5 mg/kg in diuretic-pretreated patients (5). Two hours after the administration of furosemide, urine output was assessed, and patients were classified as FST positive (urine output ≥ 200 mL within 2 hr following the application of the furosemide infusion) or FST negative (urine output < 200 mL/2 hr).

### Outcomes

The primary endpoint was the development of indications for RRT within 7 days after developing a moderate AKI (KDIGO stage 2). Indications for RRT were defined as hyperkalemia (≥ 6 mmol/L), diuretic-resistant hypervolemia, urea serum levels greater than or equal to 150 mg/dL, severe metabolic acidosis (pH ≤ 7.15), oliguria (urinary output < 200 mL/12 hr), or anuria. Secondary outcomes included the development of severe AKI (defined as KDIGO stage 3), initiation of RRT, renal recovery (serum creatinine [SCr] < 2 times of baseline value) at day 90, RRT at day 90, mortality at day 90, and major adverse kidney events_90_ (MAKE_90_) (combined endpoint consisting of mortality, RRT, and persistent renal dysfunction [defined as ≥ 2 times baseline SCr] at day 90).

### Biomarker Analyses

Urine samples were collected at the day of enrollment prior to the FST. Samples were centrifuged and supernatants were frozen in dry ice, stored at –80°C and thawed immediately prior to analysis. Urinary CCL14, urinary NGAL, and plasma dipeptidyl peptidase 3 (DPP3) were measured by enzyme-linked immunosorbent assays according to the manufacturers’ protocols (Sigma-Aldrich, an affiliate of Merck KGaA, Darmstadt, Germany; Sphingotec, Hennigsdorf, Germany; respectively) (11).

The adjudicating providers and/or RRT providers were blinded to the CCL14 results.

### Statistical Analysis

The study population was described by absolute and relative frequencies, mean and sd, where appropriate. Continuous variables were tested using Mann-Whitney *U* tests due to lacking normality. The association between categorical variables and the two groups (one or more indications vs no indications) was tested with chi-square tests.

All analyses were conducted as exploratory analyses of hypothesis generation and were not adjusted for multiple testing. All *p* values and confidence limits were two-sided and were intended to be exploratory, not confirmatory. In this exploratory sense, *p* values of less than or equal to 0.05 were considered as statistically noticeable.

To analyze the predictive power of selected combinations of biomarkers, FST and clinical covariates regarding the development of an indication for RRT, logistic regression models were fitted, corresponding receiver operating characteristic (ROC) curves were calculated and the AUC was determined. 95% CIs were reported. For optimal threshold selection, the maximum value of Youden index was determined and associated sensitivities, specificities, positive predictive values (PPVs), and negative predictive values (NPVs) were reported. To compare two nested predictive models, the likelihood-ratio test was applied. The corresponding AUCs were compared using a bootstrap test for paired data. To evaluate the additional information the larger model gives for risk classification in comparison to a reduced model, we calculated the integrated discrimination improvement (IDI) and the category-free net reclassification index (cfNRI). To compare the predictive performance of the biomarkers between both FST groups (negative/positive), AUCs were compared using a bootstrap test for unpaired data.

To identify possible clinical predictors for the primary endpoint, we performed a stepwise backward variable selection based on the *p* value of the likelihood-ratio test with a threshold of 0.1.

Descriptive statistics, statistical tests, and variable selection were performed using SPSS 21 (IBM SPSS Statistics for Windows, Version 21.0. Armonk, NY: IBM Corp.). Logistic regressions, ROC analyses and statistical tests for model comparisons were performed using *R* (Version R-4.1.2 for Windows; R Foundation for Statistical Computing, Vienna, Austria; http://R-project.org/). AUCs were compared using the function roc.test in the *R* package *pROC*. IDI and cfNRI have been calculated using the *R* package *PredictABEL* (XYZ). *p* values and 95% CIs for IDI and cfNRI are provided by the function reclassification and are determined using a z-transformation of the statistics.

## RESULTS

### Patients

**Figure 1.**
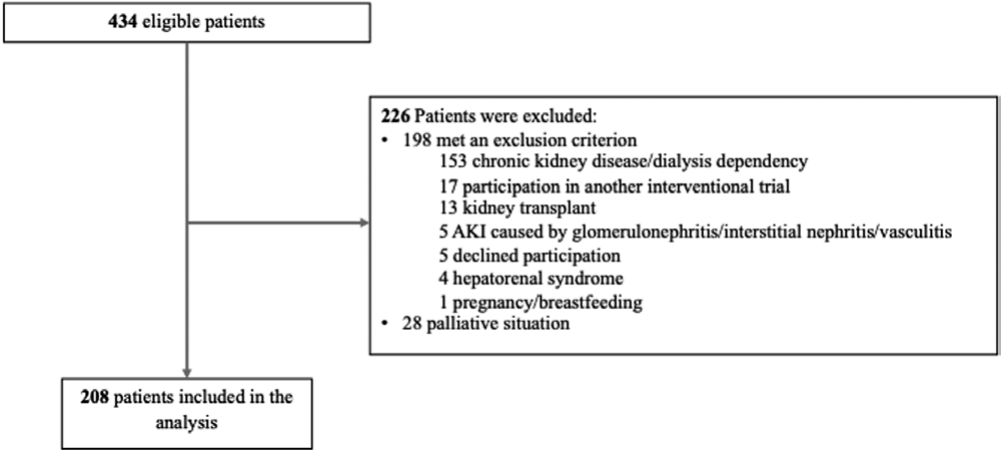
Participant flow.

Four hundred thirty-four patients were screened for eligibility, of whom 208 were included in the trial, 108 patients with a negative FST and 100 patients with a positive FST (**Fig. 1**).

Patient characteristics at baseline are shown in **Table 1**. The mean age was 70 years (sd, 12 yr), 63% were men, 84% were mechanically ventilated, and 95% received high doses of vasopressors. At inclusion, the median Sequential Organ Failure Assessment score was 12 (Q1–Q3, 9–14) and the median Acute Physiology and Chronic Health Evaluation II score 27 (Q1–Q3, 22–35). All patients were postoperative, with 57% receiving an emergency procedure. The most common procedures were cardiothoracic (49%) and general surgery procedures (16.3%) (Table 1).

**TABLE 1.** Baseline and Patient Characteristics

Characteristics	No Indication for Dialysis (*n* = 110)	Indication for Dialysis (*n* = 98)	*p*
Age, mean (sd), yr	69.6 (12.2)	69.4 (11.7)	0.46
Male sex, *n* (%)	75 (68.2)	56 (57.7)	0.12
Weight, mean (sd), kg	85.2 (22.5)	81.0 (21.5)	0.08
Sequential Organ Failure Assessment score, median (Q1–Q3)	10 (8–12)	13 (10–16)	< 0.001
Acute Physiology and Chronic Health Evaluation score, median (Q1–Q3)	26 (22–33)	31 (23–36)	0.004
Fluid balance at inclusion, median (Q1–Q3), mL	399 (–647 to 1,427)	1,476 (160–3,044)	< 0.001
Creatinine at inclusion, mean (sd), mg/dL	1.9 (1.4)	2.5 (0.9)	< 0.001
Creatinine baseline, mean (sd), mg/dL	1.01 (0.43)	1.08 (0.47)	0.13
Comorbidities, *n* (%)			
Hypertension	81 (73.6)	61 (63.5)	0.12
Congestive heart failure	29 (26.4)	22 (22.9)	0.57
Diabetes	29 (26.4)	24 (24.7)	0.79
Chronic obstructive pulmonary disease	24 (21.8)	18 (18.6)	0.56
Chronic kidney disease	28 (25.9)	38 (40.4)	0.028
Arrhythmia	40 (36.4)	32 (33.0)	0.61
Stroke	17 (15.5)	9 (9.4)	0.19
Medication, *n* (%)			
ß-blockers	64 (58.7)	61 (64.2)	0.42
Statins	54 (49.5)	43 (44.8)	0.50
Diuretics	54 (49.5)	53 (55.2)	0.42
Angiotensin-converting enzyme inhibitors	38 (34.9)	31 (32.6)	0.74
Angiotensin receptor blockers	31 (28.4)	21 (22.1)	0.30
Surgical discipline, *n* (%)			0.12
Cardiothoracic surgery	51 (47.2)	51 (55.4)	
General surgery	17 (15.7)	17 (18.5)	
Neurosurgery	14 (13.0)	4 (4.3)	
Trauma surgery	12 (11.1)	3 (3.3)	
Obstetrics/urology	3 (2.8)	4 (4.3)	
Orthopedics	1 (0.9)	1 (1.1)	
Others	10 (9.3)	12 (13.0)	
Type of surgery, *n* (%)			0.95
Elective	45 (41.3)	38 (40.9)	
Emergency	64 (58.7)	55 (59.1)	
Furosemide stress test, *n* (%)			< 0.001
UO < 200 mL in 2 hr	28 (25.5)	80 (81.6)	
UO > 200 mL in 2 hr	82 (74.5)	18 (18.4)	
Urinary chemokine (C-C motif) ligand 14, median (Q1–Q3), ng/mL	1.34 (0.74–3.64)	6.47 (3.67–13.40)	< 0.001

UO = urine output.

### Outcomes

The primary endpoint of the study, development of one or more predefined indications for RRT, was met in 98 of 208 patients (47.1%) (71.4% oliguria [urine output < 200 mL/12 hr or anuria], 38.8% urea serum levels, 30.6% diuretic-resistant hypervolemia, 21.4% hyperkalemia, and 7.1% severe metabolic acidosis). Of 70 of 98 (71.4%) meeting the urine output less than 200 mL/12 hr/anuria criterion, 44 of 70 (62.9%) met a second indication, 19 of 70 (27.1%) were anuric, and seven of 70 (0.1%) showed a urine output of less than 200 mL in 12 hours.

The time from inclusion to development of an absolute indication was median 0 days (Q1–Q3, 0–1 d) and the time from absolute indication to initiation of RRT was median 0 days (Q1–Q3, 0–1 d).

Except for renal recovery at 90 days, all other secondary endpoints (RRT during hospital stay, ICU length of stay, hospital length of stay, mortality, RRT at day 90, occurrence of MAKE_90_) were significantly higher in patients developing a predefined indication compared with patients not developing an indication (**Table 2**; and **eTable 1**, http://links.lww.com/CCM/H319). In terms of RRT, five patients (4.5%) not developing an absolute indication received RRT, whereas 69 (71.1%) developing an absolute indication were treated with RRT. Of 29 patients not receiving dialysis, in seven patients, care was changed to palliation prior to initiating dialysis (100% died) and in 22 patients, the attending intensivist decided not to initiate dialysis (50% died).

**TABLE 2.** Outcome Parameters Grouped by Development of Absolute Indication

Outcome	No Indication (*n* = 110)	Indication (*n* = 98)	*p*
Acute kidney injury stage 3, *n* (%)	24/108 (22.2)	84/96 (87.5)	< 0.001
RRT during index hospital stay, *n* (%)	5/110 (4.5)^a^	69/98 (71.1)^b^	< 0.001
ICU length of stay, median (Q1–Q3), d	10 (5–21)	37 (14–59)	< 0.001^c^
Hospital length of stay, median (Q1–Q3), d	32 (15–66)	59 (28–90)	< 0.001^c^
Renal recovery at day 90, *n* (%)	62/80 (77.5)	27/42 (64.3)	0.14^d^
RRT at day 90, *n* (%)	0/81 (0)	3/41 (7.3)	0.036
Mortality at day 90, *n* (%)	29/110 (26.4)	56/98 (57.1)	< 0.001
Major adverse kidney events_90_, *n* (%)	48/109 (44.0)	70/98 (71.4)	< 0.001

RRT = renal replacement therapy.

a Relative indications for RRT: volume overload (3x), hyperphosphatemia (1x), and creatine kinase elevation (1x).

b Of 29 patients not receiving dialysis, in seven patients care was changed to palliative prior to initiating dialysis (all of these patients died) and in 22 patients the attending intensivist decided not to initiate dialysis of which 11 patients died. Remaining 11 patients: uremia in seven patients, diuretic-resistant volume overload in three patients, acidosis in one patient, and hyperkalemia in one patient. Four out of 11 patients showed negative response to furosemide stress test (FST) and 10 out of 11 elevated chemokine (C-C motif) ligand 14 (CCL14) levels. At 90 d, two out of 11 patients had a persistent renal dysfunction, both having suffered from uremia. In both patients, CCL14 levels were elevated but FST was only negative in one patient.

c Log rank, time-to-event analysis starting at date of enrollment censoring for death.

d Dead patients at day 90 excluded, one missing.

Stratified analyses showed that patients with a negative FST and high levels of CCL14 had significantly higher rates of secondary outcomes except for renal recovery at 90 days compared with patients with a negative FST and low CCL14 levels or patients with positive FST test (**eTables 2–4**, http://links.lww.com/CCM/H319).

### Predictive Value of FST and Biomarkers

The AUCs for predicting the development of an indication for RRT were 0.79 (95% CI, 0.74–0.85) for FST, 0.83 (95% CI, 0.77–0.89) for CCL14, 0.73 (95% CI, 0.65–0.80) for DPP3, and 0.73 (95% CI, 0.66–0.802) for NGAL. The AUC for the combination of FST and CCL14 (AUC, 0.87; 95% CI, 0.82–0.92) compared with FST (*p* < 0.001) or CCL14 (*p* = 0.03) alone was significantly higher (likelihood-ratio test *p* < 0.001, respectively).

In patients with a negative FST, the AUC for CCL14 for predicting the primary endpoint was 0.86 (95% CI, 0.77–0.94), whereas in patients with a positive FST, the AUC was 0.66 (95% CI, 0.52–0.80; *p* = 0.019) indicating a complimentary value of both parameters (**Table 3**).

**TABLE 3.** Predictive Value of Biomarkers Categorized by Furosemide Stress Test Response

Biomarker	FST Negative (UO < 200 mL/2 hr), AUC (95% CI)	FST Positive (UO > 200 mL/2 hr), AUC (95% CI)	*p* ^a^
Chemokine (C-C motif) ligand 14	0.855 (0.770–0.940)	0.658 (0.517–0.800)	0.019
Neutrophil gelatinase-associated lipocalin	0.716 (0.614–0.819)	0.718 (0.602–0.834)	0.98
Dipeptidyl peptidase 3	0.697 (0.568–0.826)	0.707 (0.572–0.843)	0.91

AUC = area under the curve, FST = furosemide stress test, UO = urine output.

a *p* for the AUC difference between FST negative and FST positive.

The optimal cutoff value (maximizing Youden index) for the prediction of the primary endpoint in FST negative patients was 2.14 ng/dL (Youden index, 0.653; sensitivity, 0.961; specificity, 0.692, PPV, 0.901; NPV, 0.857).

### Additional Predictive Ability of FST and CCL14 Over Clinical Variables

The derived clinical model identified four variables predictive of the development of RRT indications which comprised of male sex, preexisting CKD, hypertension, and positive fluid balance. The addition of FST and CCL14 results significantly improved risk prediction when added to the derived clinical model for the primary endpoint. This is supported by ROC, AUC, IDI, and cfNRI analyses (**Table 4**).

**TABLE 4.** Multivariable Logistic Regression Model Using Clinical Variables for Prediction of Indication for Dialysis Without (Reference Model) and With (New Model) Furosemide Stress Test + Chemokine (C-C Motif) Ligand 14

Variable	Reference Model^a^		New Model With FST and CCL14	
	OR (95% CI)	*p*	OR (95% CI)	*p*
Male sex	0.56 (0.29–1.06)	0.08	0.61 (0.27–1.35)	0.22
Chronic kidney disease	1.88 (0.97–3.70)	0.06	1.15 (0.55–2.93)	0.57
Hypertension	0.54 (0.27–1.06)	0.07	0.49 (0.20–1.13)	0.10
Fluid balance	1.0004 (1.0002–1.0006)	< 0.001	1.0002 (1.0000–1.0005)	0.05
FST	—	—	8.42 (3.88–19.29)	< 0.001
CCL14	—	—	1.15 (1.08–1.24)	< 0.001
Comparison of Models		Value	95% CI	*p*
AUC (reference model)		0.714	0.640–0.789	< 0.001
AUC (new model)		0.873	0.823–0.924	< 0.001
Integrated discrimination improvement		0.277	0.212–0.324	< 0.001
Category-free net reclassification index		1.056	0.814–1.297	< 0.001

AUC = area under the curve, CCL14 = chemokine (C-C motif) ligand 14, FST = furosemide stress test, OR = odds ratio.

a The following additional clinical variables were included in an initial version of the multivariable logistic regression model but were removed during stepwise variable selection: age, body weight, diabetes, and vasopressors. Dashes indicate variables were not included in the reference model.

## DISCUSSION

Although several randomized controlled trials have examined the optimal timing for initiation of RRT in critically ill patients with AKI, the answer remains elusive (2–4, 12). Given that commencing RRT based solely on AKI staging criteria results in unnecessary treatment in approximately 50% of patients, and in individuals who receive treatment “late” a higher mortality is observed identification of the time where initiating RRT is ideal has great clinical value (2, 3). Currently, no tools are available that can accurately identify patients optimal timing and consequently the use of RRT is highly variable given that it is often based on the decision of the treating physician. In the STandard versus Accelerated Initiation of Renal Replacement Therapy in Acute Kidney Injury trial (STARRT-AKI), clinician equipoise was used to stipulate enrollment of patients with a stage 2 AKI (4). Approximately 30% of the patients were enrolled in which the clinicians were unsure whether the patients would benefit from RRT (4). In the late group, in 25% of the patients, RRT had to be started within 19 hours and in 24% of the patients, the start could be delayed up to 72 hours or longer. Thirty-eight percent of the patients did not receive RRT at all. Thus, physician perceptions on the need for RRT are imperfect and would benefit from diagnostic or prognostic augmentation. The Artificial Kidney Initiation in Kidney Injury 2 Trial (AKIKI2) tested the hypothesis whether a more-delayed initiation strategy would result in more RRT-free days compared with a delayed strategy (13). The study showed that there was higher risk of mortality at 60 days in the more-delayed group. These controversial trial results sparked a discussion on when to start RRT and whether a “wait and see” approach is preferable over an early initiation of RRT. However, it is still unknown which patients would benefit from an initial waiting approach and when this watchful watching can turn harmful necessitating a change in management.

Other studies have investigated the predictive ability of FST and CCL14 for the development of progression of AKI. In a multicenter study including 77 critically ill patients with early AKI, the AUC for the progression to Acute Kidney Injury Network stage 3 was 0.87 (5). The ideal cutoff during the first 2 hours following FST was a urine output of less than 200 mL. These data were further validated in an international cohort of 96 patients with 200ml in 2 hours post FST providing a sensitivity of 74% and specificity of 90% for progression to stage 3 AKI. The 2-hour urine output provided an identical AUC of 0.87 for the progression to stage 3 in this cohort (14). In one small cohort of 11 patients, the predictive ability of FST in combination with biomarkers (NGAL, tissue inhibitor of metalloproteinase-2 × insulin-like growth factor binding protein 7) was analyzed for RRT receipt (15). The combination of FST plus NGAL improved the AUC from 0.86 for FST alone to 0.88 for FST + NGAL but without significance (*p* = 0.35). Of note is that again, there is a discrepancy between receiving and needing RRT.

CCL14 has been studied in the ICU setting as well as in a cardiac surgical cohort with a high predictive ability, again for the primary endpoint progression of AKI (8, 9, 16). However, not all patients developing a progression of AKI develop an indication for RRT, which was the purpose of this study. It is important to predict the need for RRT because the current standard of care is quite variable and initiation of RRT often depends on the discretion of the treating physician. As AKI is heterogenous in etiology, we believe that the combination of tests helps to improve the predictive value; hence, we combined the FST with CCL14. We also used the Youden index to determine the cutoff value with the best predictive value in our cohort. The different cutoff values in other published studies can be explained by investigating different patient cohorts and/or by predicting different endpoints (8, 9, 16).

Here, we provide the first evidence that a combination of the FST with the novel biomarker CCL14 has a higher predictive value for the development of one or more predefined indications for RRT than the tests in isolation. Application of such a combination may obviate the variability in application of RRT in critically ill patients with AKI with the associated high mortality rate and resource utilization (1). Furthermore, these results could also help in designing future trials addressing the optimal time point of initiating RRT. To our knowledge, this is the first study that combines a renal stress test with novel renal biomarkers and demonstrated the ability of predicting one or more predefined indications for RRT.

In a pilot trial that randomized FST negative patients into early and late initiation of RRT, no difference in 28-day mortality could be demonstrated (6). Of 60 patients randomized to the late group, 25% spontaneously recovered renal function showing that the FST test alone is not sufficient for detecting patients who will need RRT. Patient-individualized strategies using novel biomarkers have been shown to be effective for the prevention of AKI (17–19). This leads to the hypothesis that a similar strategy may also be useful for determining initiation of RRT. Here, we demonstrate that critically ill patients with a negative FST and high biomarker levels have a high risk for developing an indication for RRT. Therefore, the combination of these two tests may identify patients who are likely to benefit most from RRT whilst potentially avoiding escalation for others.

The study is not without limitations. First, this is a pilot single-center observational trial where selection bias cannot completely be controlled for. The intention was to gain a first insight into the possibility of combining the two tests (FST and CCL14) and detect patients who progress to RRT-dependent AKI. Second, we only included surgical patients, which represents only a subpopulation of hospitalized severe AKI. However, given the diversity of surgical patients, the underlying sources of AKI was not uniform across our entire cohort, speaking to the broader applicability of this work. Third, not all patients developing an absolute indication for RRT received treatment. Besides the fact that treatment plan was changed for some of these patients, this problem is representative for daily routine care. A clear profile for those patients not receiving treatment was not detectable. Future trials have to address the question whether the test combination has also such a good predictive value in AKI of various pathophysiologies (20, 21).

In conclusion, we found that the combination of the FST with the novel biomarker CCL14 predicts the development of indications for RRT. Future trials are warranted to investigate whether a patient-individualized strategy for the initiation of RRT is feasible.

## ACKNOWLEDGMENT

We acknowledge the support by the Open Access Publication Fund of the University of Münster, Germany.

## Supplementary Material
