# Supplementary material for: Predicting the Development of Renal Replacement Therapy Indications by Combining the Furosemide Stress Test and Chemokine (C-C Motif) Ligand 14 in a Cohort of Postsurgical Patients
Source: Crit Care Med. 2023 Mar 29;51(8):1033–42. doi: 10.1097/CCM.0000000000005849 (PMC10335738; doi:10.1097/CCM.0000000000005849)
Supplement: Supplementary file 1 [file ccm-51-1033-s001.docx]

**Supplementary Material for**

**Predicting the development of renal replacement therapy indications by combining the furosemide stress test and CCL14 in a cohort of postsurgical patients**

**TABLE OF CONTENTS**

**CONTENT PAGE**

[eTable 1: Modality of RRT and renal recovery 2](#_Toc123897996)

[eTable 2: Outcome parameters grouped by FST 3](#_Toc123897997)

[eTable 3: Outcome parameters grouped by CCL14 4](#_Toc123897998)

[eTable 4: Outcome parameters grouped by FST and CCL14 5](#_Toc123897999)

## eTable 1: Modality of RRT and renal recovery

| Outcome | No indication (n=5) | Indication (n=69) | Renal recovery at day 90 (n=20) |
| --- | --- | --- | --- |
| Modality of RRT, No. (%) |  |  |  |
| CRRT | 4 (80) | 46 (66.7) | 12 (60) |
| SLEDD | 1 (20) | 4 (5.8) | 2 (10) |
| IHD | 0 | 1 (1.4) | 0 (0) |
| CRRT+SLEDD | 0 | 13 (18.8) | 4 (20) |
| CRRT+IHD | 0 | 2 (2.9) | 1 (5) |
| CRRT+SLEDD+IHD | 0 | 3 (4.3) | 1 (5) |

Abbreviations: CRRT, continuous renal replacement therapy; IHD, intermittent hemodialysis; RRT, renal replacement therapy; SLEDD, slow low efficient daily dialysis.

## eTable 2: Outcome parameters grouped by FST

| Outcome |  | FST positive  (n=100) | FST negative  (n=108) | *P*-value |
| --- | --- | --- | --- | --- |
| AKI stage 3, No. (%) |  | 27/97 (27.8) | 81/107 (52.9) | <0.001 |
| Dialysis during index hospital stay, No. (%) |  | 11/100 (11.0) | 63/108 (58.3) | <0.001 |
| ICU length of stay, median (Q1, Q3), days |  | 10 (5, 22) | 22 (10, 52) | <0.001^a^ |
| Hospital length of stay, median (Q1, Q3), days |  | 33 (15, 70) | 49 (28, 87) | 0.068^a^ |
| Renal recovery at day 90, No. (%) |  | 55/72 (76.4) | 34/50 (68.0) | 0.305^b^ |
| Dialysis at day 90, No. (%) |  | 0/74 (0) | 3/48 (6.3) | 0.059 |
| Mortality at day 90, No. (%) |  | 27/100 (27) | 58/108 (53.7) | <0.001 |
| MAKE_90_, No. (%) |  | 44/99 (44.4) | 74/108 (68.5) | <0.001 |

Abbreviations: AKI, acute kidney injury; ICU, intensive care unit; MAKE, major adverse kidney events

^a^ Log rank, time-to-event analysis starting at date of enrolment censoring for death

^b^ Dead patients at day 90 excluded, 1 missing

## eTable 3: Outcome parameters grouped by CCL14

| Outcome |  | CCL14≤2.44^a^  (n=85) | CCL14>2.44 (n=114) | *P*-value |
| --- | --- | --- | --- | --- |
| AKI stage 3, No. (%) |  | 24/85 (28.2) | 79/110 (71.8) | <0.001 |
| Dialysis during index hospital stay, No. (%) |  | 10/85 (11.8) | 59/114 (51.8) | <0.001 |
| ICU length of stay, median (Q1, Q3), days |  | 13 (5, 36) | 17 (7, 47) | 0.068^b^ |
| Hospital length of stay, median (Q1, Q3), days |  | 42 (17, 71) | 38 (20, 84) | 0.87^b^ |
| Renal recovery at day 90, No. (%) |  | 42/56 (75.0) | 44/62 (71.0) | 0.62^c^ |
| Dialysis at day 90, No. (%) |  | 0/56 (0) | 3/62 (4.8) | 0.25 |
| Mortality at day 90, No. (%) |  | 28/85 (32.9) | 52/114 (45.6) | 0.071 |
| MAKE_90_, No. (%) |  | 43/84 (51.2) | 69/114 (56.6) | 0.19 |

Abbreviations: AKI, acute kidney injury; ICU, intensive care unit; MAKE, major adverse kidney events

^a^ Best CCL14 - cutoff for predicting absolute indication in total cohort.

^b^ Log rank, time-to-event analysis starting at date of enrolment censoring for death

^c^ Dead patients at day 90 excluded, 1 missing

## eTable 4: Outcome parameters grouped by FST and CCL14

| **Outcome** |  | **FST pos. &  CCL14 ≤ 2.14^a^ (n=51)** | **FST pos. &**  **CCL14 > 2.14**  **(n=46)** | **FST neg. &**  **CCL14 ≤ 2.14**  **(n=21)** | **FST neg. &**  **CCL14 > 2.14**  **(n=81)** | ***P*-value** |
| --- | --- | --- | --- | --- | --- | --- |
| AKI stage 3, No. (%) |  | 13/51 (25.5) | 14/43 (32.6) | 7/21 (33.3) | 69/80 (86.3) | <0.001 |
| Dialysis during index hospital stay, No. (%) |  | 7/51 (13.7) | 4/46 (8.7) | 1/21 (4.8) | 57/81 (70.4) | <0.001 |
| ICU length of stay, median (Q1, Q3), days |  | 9 (5, 22) | 10 (4, 20) | 10 (6, 22) | 37 (11, 59) | <0.001^b^ |
| Hospital length of stay, median (Q1, Q3), days |  | 40 (15, 71) | 22 (14, 51) | 44 (14, 59) | 52 (28, 90) | 0.028^b^ |
| Renal recovery at day 90, No. (%) |  | 29/38 (76.3) | 25/32 (78.1) | 9/12 (75.0) | 23/36 (63.9) | 0.54^c^ |
| Dialysis at day 90, No. (%) |  | 0/39 (0) | 0/33 (0) | 0/11 (0) | 3/35 (8.6) | 0.063 |
| Mortality at day 90, No. (%) |  | 13/51 (25.5) | 13/46 (28.3) | 9/21 (42.9) | 45/81 (55.6) | 0.002 |
| MAKE_90_, No. (%) |  | 22/51 (43.1) | 20/45 (44.4) | 13/21 (61.9) | 57/81 (70.4) | 0.005 |

Abbreviations: AKI, acute kidney injury; ICU, intensive care unit; MAKE, major adverse kidney events

^a^ Best CCL14 - cutoff for predicting absolute indication in FST negative cohort.

^b^ Log rank, time-to-event analysis starting at date of enrolment censoring for death

^c^ Dead patients at day 90 excluded, 1 missing
